# Osmotic Imbalance, Cytoplasm Acidification and Oxidative Stress Induction Support the High Toxicity of Chloride in Acidophilic Bacteria

**DOI:** 10.3389/fmicb.2019.02455

**Published:** 2019-10-29

**Authors:** Javier Rivera-Araya, Andre Pollender, Dieu Huynh, Michael Schlömann, Renato Chávez, Gloria Levicán

**Affiliations:** ^1^Laboratory of Basic an Applied Microbiology, Department of Biology, Faculty of Chemistry and Biology, University of Santiago, Santiago, Chile; ^2^Environmental Microbiology, Institute of Biosciences, TU Bergakademie Freiberg, Freiberg, Germany

**Keywords:** *Leptospirillum* spp., acidophiles, chloride, bioleaching, oxidative stress, osmolarity, compatible solutes

## Abstract

In acidophilic microorganisms, anions like chloride have higher toxicity than their neutrophilic counterparts. In addition to the osmotic imbalance, chloride can also induce acidification of the cytoplasm. We predicted that intracellular acidification produces an increase in respiratory rate and generation of reactive oxygen species, and so oxidative stress can also be induced. In this study, the multifactorial effect as inducing osmotic imbalance, cytoplasm acidification and oxidative stress in the iron-oxidizing bacterium *Leptospirillum ferriphilum* DSM 14647 exposed to up to 150 mM NaCl was investigated. Results showed that chloride stress up-regulated genes for synthesis of potassium transporters (*kdpC* and *kdpD*), and biosynthesis of the compatible solutes (hydroxy)ectoine (*ectC* and *ectD*) and trehalose (*otsB*). As a consequence, the intracellular levels of both hydroxyectoine and trehalose increased significantly, suggesting a strong response to keep osmotic homeostasis. On the other hand, the intracellular pH significantly decreased from 6.7 to pH 5.5 and oxygen consumption increased significantly when the cells were exposed to NaCl stress. Furthermore, this stress condition led to a significant increase of the intracellular content of reactive oxygen species, and to a rise of the antioxidative cytochrome *c* peroxidase (CcP) and thioredoxin (Trx) activities. In agreement, *ccp* and *trx* genes were up-regulated under this condition, suggesting that this bacterium displayed a transcriptionally regulated response against oxidative stress induced by chloride. Altogether, these data reveal that chloride has a dramatic multifaceted effect on acidophile physiology that involves osmotic, acidic and oxidative stresses. Exploration of the adaptive mechanisms to anion stress in iron-oxidizing acidophilic microorganisms may result in new strategies that facilitate the bioleaching of ores for recovery of precious metals in presence of chloride.

## Introduction

When cells are exposed to NaCl, the water is drawn to the extracellular space, with the subsequent decrease of cell turgor pressure, cell wall strain, cytoplasmic membrane tension and dehydration of the cytoplasm (Roberts, 2005; Wood, 2015). To prevent these phenomena, bacteria display an osmoadaptative response which consists of active uptake or *de novo* synthesis of solutes in the cytoplasm; whereby the water flux is attenuated. These solutes include inorganic ions (often K^+^) as a first response mechanism, and organic molecules nominated “compatible solutes” as a long-term response to the stress condition (Csonka, 1989**;** Sleator and Hill, 2001; Wood, 2011, 2015). In *Escherichia coli* K^+^-uptake and its homeostasis are regulated by the constitutively expressed Kup and Trk transporters, and the osmotically regulated Ygg and Kdp transporters (Csonka, 1989; Ito et al., 2009). On the other hand, compatible solutes are highly soluble organic molecules, without net charge at physiological pH that do not impair native conformation and function of biomolecules, and therefore can be accumulated in high concentrations (up to molar) (Zhao et al., 2007; Wood, 2015). The mechanisms of compatible solute accumulation under high osmotic pressure in ***E. coli*** consist of synthesis of trehalose or glycine betaine (via OtsAB and BetAB pathways, respectively); or uptaking choline, betaine and other organic molecules by ProP, ProU, BetT, and BetU transporters, respectively (Wood, 2015). Other compatible solutes present in prokaryote also include alanine, glutamine, sucrose, mannosylglycerate, sorbitol, and mannitol, among others (Empadinhas and da Costa, 2008).

Acidophilic iron-oxidizing microorganisms play a key role in bioleaching of sulfide minerals and metal extraction in both natural and man-made environments (Bonnefoy and Holmes, 2011; Johnson and Schippers, 2017). These microorganisms use ferrous iron as primary energy source, thus through Fe^2+^ oxidation, they are able to re-generate ferric (Fe^3+^) iron, which is assumed to have a relevant role as oxidant for dissolving the minerals. The process results in accumulation of ferric iron and other soluble metals in acid drainages derived from mines or rocks (Johnson, 2014). In bioleaching industrial environments, cations can additionally be accumulated due to the source water, recycling of process waters, evaporation, and because of gangue mineral dissolution (Rea et al., 2015). Iron-oxidizing acidophilic microorganisms have an unusual tolerance to cations, but they are extremely sensitive to anions (except sulfate) (Alexander et al., 1987; Suzuki et al., 1999; Rea et al., 2015). Particularly, chloride has proven to be highly inhibitory for a number of acidophiles such as *Acidithiobacillus ferrooxidans*, *Acidithiobacillus caldus*, *Leptospirillum ferrooxidans*, *Leptospirillum ferriphilum*, *Sulfobacillus thermosulfidooxidans*, and also for mesophilic microbial consortia in which *Leptospirillum* was found to be dominant (Simmons and Norris, 2002; Marhual et al., 2008; Gahan et al., 2009, 2010; Wang et al., 2012; Guo et al., 2014; Galleguillos et al., 2018; Huynh et al., 2019). It is important to note that in these studies, the counter-ions sodium and potassium did not exert a toxic effect on the physiology of the acidophilic microorganisms since the Na_2_SO_4_ and K_2_SO_4_, do not inhibit the growth and the iron oxidation activity (Kieft and Spence, 1988; Suzuki et al., 1999). Studies using *A. ferrooxidans* as acidophile model have allowed to establish that osmotic imbalance induced by chloride and other anions disrupt the positive internal membrane potential, and lead to the influx of protons and acidification of the cytoplasm (Alexander et al., 1987; Zammit and Watkin, 2016).

Until now, no extremely halotolerant acidophilic microorganisms have been described. However, moderately halotolerant *Sulfobacillus thermosulfidooxidans* Cutipay (3 g L^–1^ NaCl), *L. ferriphilum* Sp-Cl (12 g L^–1^ NaCl), *Acidihalobacter* (*Thiobacillus*) prosperus DSM 5130 (35 g L^–1^ NaCl), *Acidihalobacter aeolianus* sp. nov. (=DSM 14174), *Acidihalobacter ferrooxidans* sp. nov. (=DSM14175) (45 g/L NaCl), and *Alicyclobacillus* sp. S09 have been isolated (Huber and Stetter, 1989; Bobadilla-Fazzini et al., 2014; Issotta et al., 2016; Dopson et al., 2017; Huynh et al., 2017; Khaleque et al., 2019). The mechanisms that these or other acidophilic microorganisms use to withstand the osmotic stress are recently being investigated. However, evidence derived from various studies has so far shown that they use canonical mechanisms of protection. For example, *Acidiphilium cryptum* synthesizes hydroxyectoine in response to moderate osmotic stress with NaCl and Al_2_(SO_4_)_3_ (Moritz et al., 2015). *Leptospirillum* spp. use accumulation of potassium and of the compatible solutes (hydroxy)ectoine and trehalose, as strategy to tolerate high osmolarity (Parro et al., 2007; Mosier et al., 2013; Galleguillos et al., 2018). *At. ferrooxidans* uses proline and betaine as osmoprotectans (Kieft and Spence, 1988). Synthesis of proline and DNA binding proteins have also been described as a NaCl adaptation mechanism for *At. caldus* (Guo et al., 2014). Furthermore, a proteomic study conducted to evaluate the response of the moderately halotolerant *Ac. prosperus* to chloride stress suggested a response based on the synthesis and uptake of ectoine (Dopson et al., 2017). In addition, this microorganism also seems to have developed a more specific adaptive response that involves changes in amino acid composition of the rusticyanin protein. When compared with rusticyanin from non-halotolerant *At. ferrooxidans*, rusticyanin from *Ac. prosperus* displayed a more negative surface potential which is predicted to contribute to protecting the environment of the copper ion that makes up the redox center of the protein (Dopson et al., 2017).

Interestingly, proteomic studies carried out in *Ac. prosperus* also suggested an increased iron oxidation activity. In acidophilic microorganisms, respiration is one of the principal mechanisms to keep the intracellular pH close to neutrality, as protons are extruded to the exterior or consumed at the cytoplasmic level during NAD(P) + and O_2_ reduction to form NAD(P)H and H_2_O, respectively (Quatrini et al., 2009; Levicán et al., 2012). However, an increase in the respiratory rate involves a higher probability of electron leakage, and therefore, reactive oxygen species (ROS) that are toxic for the cell may be produced (Ziegelhoffer and Donohue, 2009). These theoretical considerations suggested that exposure to anions, and in particular to chloride could induce a condition of oxidative stress that would partly explain their highly toxic effect. Although, in recent years, the response to oxidative stress has been studied in some acidophiles (Ram et al., 2005; Norambuena et al., 2012; Contreras et al., 2015; Ferrer et al., 2016a; Zapata et al., 2017; Bellenberg et al., 2019), a relationship between saline stress and the response to oxidative stress has not been established yet.

In this work, we addressed the hypothesis that high chloride susceptibility of iron-oxidizing acidophilic microorganisms is due to a widespread multifactorial phenomenon that involves osmotic imbalance, acidification of the cytoplasm and oxidative stress induction. Thus, parameters characteristic for each of these conditions were evaluated in *L. ferriphilum* DSM 14647.

## Materials and Methods

### Bacterial Strains and Growth Conditions

*Leptospirillum ferriphilum* DSM 14647 was grown in DMSZ 882 medium pH 1.8 supplemented with ferrous sulfate (20 g/l). Bacterial growth was carried out at 180 rpm and 37°C.

### Determination of Relative Levels of RNA

#### RNA Isolation and cDNA Synthesis

*Leptospirillum ferriphilum* was grown until late exponential phase. Cells were harvested by centrifugation at 8,000 × *g* for 15 min and washed once with acid water (10 mM H_2_SO_4_) and twice with 10 mM sodium citrate pH 7.0. Washed cells were suspended in DSMZ 882 medium and incubated with 100 mM NaCl for the times indicated. Cells were collected by centrifugation at 8,000 × *g* for 10 min, and washed twice with 10 mM sodium citrate pH 7.0. RNA was isolated using the RNeasy Mini Kit (Qiagen). DNA was removed by DNase I treatment (New England, Biolabs) according to the manufacturer’s instructions. cDNA synthesis was carried out with the AffinityScript qPCR cDNA Synthesis kit (Agilent Technologies). The reaction mixture of 20 μl contained First Strand master mix, 0.1 μg/μl random primers, Affinity Script RT/RNase Block enzyme mixture and 1 μg of RNA. The synthesis was carried out at 25°C for 5 min and after that at 42°C for 15 min. Then the enzyme was inactivated at 95°C for 5 min. cDNA was stored at −80°C until further use.

#### Quantitative PCR Reaction

Primers for qPCR reactions (Table 1) were designed using the available gene sequences of *L. ferriphilum* (Cárdenas et al., 2014). The KAPA SYBR FAST qPCR kits (Kapabiosystems) was used for qPCR amplification according to the manufacturer’s instructions. The qPCR conditions were an initial denaturation at 95°C for 5 min, followed by 40 cycles of denaturation (95°C for 30 s), annealing (60°C for 20 s) and extension (72°C for 10 s). All these reactions were performed in a StepOne Real-Time PCR system (Applied Biosystems). The relative abundance of each gene versus a constitutively expressed gene (*rrsB*) by the comparative Ct (ΔΔCt) method was determined.

**TABLE 1:** Primers used for RT-qPCR.

**Gene**	**Gene product**	**Primer (5′–3′)**	**Amplicon (bp)**
*ectC*	Ectoine synthase	(F)ACGACCATCTACGCCAATACC	93
		(R)TGTCTCGACCTCTCCTTCTCC	
*ectD*	Ectoine hydroxylase	(F)AGCTGTTCCATCCTCCTGA	94
		(R)ACGCCATACTCCTGTTTTCG	
*proP*	L-Proline glycine betaine transporter	(F)TCCTTTTCCTTGCCCGTCT	109
		(R)GCCATTCTCCCTCTTCTCTTGT	
*treZ*	Malto-oligosyltrehalose trehalohydrolase	(F)TCCGACGACTTCCACCAT	131
		(R)GCAAACTGTCCCTGATAGACG	
*treS1*	Trehalose synthase	(F)GTATCAGGACCACAAGGACGA	101
		(R)CGACATCCAGATTGAACCAGT	
*treS2*	Trehalose synthase	(F)GGTGACGATGCTGTTTTCCT	132
		(R)CGACGAAGCGACAAGATAGTG	
*otsB*	Trehalose-6-phosphate phosphatase	(F)GACATTCCTCCAGGCAAACA	142
		(R)ACTTCCTCCGCTTGCTTCTT	
*kdpC*	Potassium-transporting ATPase	(F)CGGGTCGAGTCCTCTTCCTTA	126
		(R)GGCAGTCCGTTTTCTTTTCG	
*kdpD*	Osmosensitive K^+^ channel histidine kinase	(F)GCTTCTGGTCCTTCATCTTCG	114
		(R)ACATCTTCCCTCCATTTTCG	
*trkA1*	Potassium channel	(F)CTTTTGGTGACGCCTTCTTC	93
		(R)CAGGGCAATAATGGACAGC	
*trkA2*	Potassium channel	(F)GTCCCGAAAACCGATGATG	124
		(R)ACAGATGCCGCACACGAT	
*yggT*	Integral membrane protein signaling osmotic stress	(F)CTCGCCGGATCCCTACAAT	95
		(R)GCTTTTCGGGAGGAACCAGT	
*ccp (yhjA)*	Cytochrome *c* peroxidase	(F)ATGCCGCCTACTTTCCTCT	106
		(R)TAGTTGGGGTTAGCCATTTCC	
*trx1*	Thioredoxin	(F)TCGGAAGAGTATAAAGGCAAGG	106
		(R)AAAACATCAGAGTGGGAATGC	
*trx4*	Thioredoxin	(F)CTGGAAATCCCTGAGAAACG	105
		(R)GGAACCGACTGAATGGAGTG	
*trx6*	Thioredoxin	(F)TGGATGAAAACCCCTACACC	104
		(R)ATAGGCACCCACCAGTCG	
*rrsB*	RNA ribosomal 16S	(F)ACGGGTGAGTAGACATGGG	105
		(R)GGTAGGGTGCAAACGGG	

*F, forward; R, reverse.*

### Determination of Intracellular Concentration of Compatible Solutes

#### Metabolite Extraction

The metabolite extraction was performed according to Mosier et al. (2013), with some modifications. Briefly, three replicates with approximately 75 mg (wet weight) of biomass were used from each sample and were extracted in 700 μl of methanol-isopropanol-water (3:3:2 [vol/vol/vol]). Samples were then sonicated at maximum amplitude for 10 min in steps of 30 s and further centrifuged for 10 min at 18,000 × *g*. The supernatant was transferred to a clean tube, to which 5 mg of ionic exchange resin (BioRex RG 50l-XB) were added, and the mixture was shaken at 600 × *g* for 30 min at room temperature. Then, samples were centrifuged for 5 min at 15,000 × *g*, the supernatant was taken and saccharose was added to 500 μM final concentration as an internal standard for GC-MS analysis. Finally, these mixtures were dried using a SpeedVac (typically 4–8 h). Extraction blank controls also followed this complete procedure precisely, which began by adding the extraction solvent to the empty tubes.

#### Analysis of Intracellular (Hydroxy)Ectoine Content

The compatible solutes ectoine and hydroxyectoine were quantified by HPLC analysis, using an Ultimate 3000-2015 HPLC (Thermo Scientific) system with a 250 mm × 4.6 mm Hypurity Aquastar C-18 column with particle size of 5 μm (Thermo Scientific). Chromatography was performed with a gradient of two solutions as mobile phase, eluent A (0.8 mM KH_2_PO_4_/6.0 mM Na_2_HPO_4_, pH 7.6) and eluent B (acetonitrile), at a flow rate of 1.0 ml/min at 25°C. The presence of compatible solutes was monitored at 215 nm by a UV/VIS detector. The retention times of ectoine and hydroxyectoine were determined using commercially available compounds (purity ≥95%, Sigma-Aldrich). Intracellular ectoine and hydroxyectoine content was calculated as nanograms per mg of wet biomass, using a calibration curve.

#### Analysis of Intracellular Trehalose Content

The trehalose content was quantified by gas chromatography associated with mass spectrometry (GC-MS) on samples derivatized with *N-*methyl*-N*-(trimethylsilyl)trifluoroacetamide (MSTFA) according to Medeiros and Simoneit (2007) with some modifications. Briefly, dry extracts were suspended in 200 μl of pyridine, 25 μl of MSTFA were added and the mixture were heated at 60°C for 1 h. After that, they were centrifuged at maximum speed for 5 min at room temperature and the supernatants were analyzed.

Aliquots of 1 μl of silylated total extracts, as w ell as standard solutions of 500 μM trehalose/saccharose, were analyzed within 24 h, using a TRACE 1310 gas chromatograph interfaced with an (ISQ) mass-selective detector (GC–MS). A TRACE ^T*M*^ TR-5 capillary column (30 m × 0.25 mm I.D. and film thickness of 0.25 μM (Thermo Scientific) was used with helium (Airgas) as the carrier gas at a constant flow rate of 1.3 ml min^–1^. The injector and MS source temperatures were maintained at 280 and 230°C, respectively. The column temperature program consisted of injection at 65°C, held for 2 min, temperature increase from 6°C min**^–^**^1^ to 300°C, followed by an isothermal hold at 300°C for 8 min. The MS was operated in the electron impact mode with ionization energy of 70 eV. The measurement was set to 361 m/z for 5 min.

Data were acquired and processed with the Xcalibur 2.2 software. Compound identification was performed by comparison with the chromatographic retention characteristics and mass spectra of authentic standards, reported mass spectra and the mass spectral library of the GC–MS data system. Compounds were quantified using the total ion current (TIC) peak area converted to compound mass using calibration curves of internal standards with 500 μM saccharose. Procedural blanks were run in sequence on the samples in order to monitor significant background interferences.

### Determination of Intracellular pH (pH_in_)

Intracellular pH was determined by the pH rodo Green (Life Technologies) fluorescent probe according to the manufacturer’s instructions. Briefly, cells derived from the different treatments were centrifuged at 8,000 × *g* for 15 min, washed and resuspended in staining solution (pH rodo Green and Power Load, dissolved in 10 mM HEPES buffer pH 7.4). These suspensions were incubated for 30 min at 37°C in darkness under agitation at 180 rpm. Subsequently, the cells were washed in the same 10 mM HEPES buffer and incubated with 10 mM each valinomycin/nigericin solution at 37°C for 10 min. Finally, the fluorescence of 100 μl of each sample was measured using excitation/emission wavelengths of 509 and 533 nm, respectively. The relative fluorescence values obtained were related to pH by means of the pH calibration kit (Life Technologies, United States). The assay was performed in triplicate.

### Oxygen Consumption

The rate of oxygen consumption was determined by means of optodes (Fibox 3, PreSens-Precision Sensing GmbH, Regensburg, Germany) (Giebner et al., 2017). In short, fresh iron-grown cultures of *L. ferriphilum* DSM 14647 were harvested by centrifugation at 8,000 × *g* for 15 min and, after removal of supernatant, a volume of 0.1 ml resuspended cells was added to a 3 ml measuring cuvette containing 2.6 ml of DSMZ 882 culture medium (pH 1.8) with increasing NaCl concentrations, ranging from 0 to 180 mM NaCl. Afterward, 0.15 ml of ferrous iron solution were added into the cuvette to give 20 g/l final concentration and the suspension was mixed cautiously. The cuvette was then carefully closed with a glass lid. An oxygen sensing optode spot had previously been embedded inside the measuring cuvette. Fiber-optics located outside of the cuvette on the opposite side of the oxygen sensor spot was connected with a 4-channel fiber–optics oxygen meter (Firesting O_2_), also equipped with a receptacle for a temperature sensor. The optode signal was evaluated using the software Pyro Oxygen Logger. Noticeably, due to the strong temperature dependence of fluorescence, measurements had to be made in a thermostated cabinet (UVP Hybridizer HB-1000). Optode measurements were done in triplicate.

### Determination of ROS Levels

The oxidant-sensitive probe H_2_DCFDA (2’,7’-dichlorodihydrofluorescein diacetate) (Davidson et al., 1996) was used to determine the intracellular level of total ROS according to Ferrer et al. (2016b). For ROS determination, 300 ml of cell culture were centrifuged and washed with 10 mM sodium citrate pH 7.0 and incubated for 30 min in 100 mM potassium phosphate pH 7.4, containing 10 μM final concentration of H_2_DCFDA (from a 1 mM stock solution dissolved in dimethyl sulfoxide). After washing, the cells were suspended in 100 mM potassium phosphate pH 7.4, disrupted by sonication, and centrifuged at 18,000 × *g* for 20 min. Aliquots of cell extracts (100 μl) were obtained and the fluorescence intensity was measured using a fluorescence reader (Synergy HT, BioTek) and excitation at 498 nm. Emission values recorded at 522 nm were normalized to the respective protein concentration. Protein concentration was determined as described by Bradford (1976). Since incubation under neutral pH conditions could induce a detrimental effect on physiology of acidophilic bacteria, to test cell viability, a control incubating *L. ferriphilum* DSM 14647 in 100 mM potassium phosphate buffer pH 7.4 with or without the probe and re-inoculating into fresh medium was performed.

### Antioxidant Protein Activities

Antioxidant activities were measured in whole-cell extracts prepared according to Ferrer et al. (2016b). Bacterial extracts were prepared by ultrasonic disruption in buffer containing 30 mM Tris–HCl pH 8.0, 30 mM NaCl, 1 mM dithiothreitol, followed by centrifugation for 15 min at 30,000 × *g* at 4°C. As a negative control, the activities of a protein extract inactivated at 65°C for 15 min were followed.

#### Thioredoxin (Trx) Activity

Thioredoxin activity was assayed by the reduction of disulfides of free chain insulin B by dithiothreitol and measured spectrophotometrically at 650 nm as turbidity formation from the protein precipitation. The assay was carried out at room temperature with minor modifications as described by Norambuena et al. (2012).

#### Cytochrome *c* Peroxidase (CcP) Activity

Cytochrome *c* Peroxidase activity was assayed as described by Yonetani and Ray (1966). In summary, 50 mg of horse heart cytochrome *c* (Merck) were dissolved in 2 ml of 10 mM potassium phosphate pH 7.0 and 1 mM EDTA. To reduce ferricytochrome *c*, the reaction mixture was incubated with 10 mM sodium dithionite for 2 min. The salt excess was removed by gel filtration in Micro Bio-Spin columns (BioRad) packed with Bio-GelP6 (molecular exclusion limit of 1–6 kDa) (BioRad). Reduced cytochrome *c* was estimated spectrophotometrically at 550 nm. An aliquot of 10 μl was mixed with 490 μl of phosphate buffer pH 7.0 and absorbance was measured at 550 nm. The absorbance of a ferricyanide-oxidized cytochrome *c* was also determined. The percentage of cytochrome *c* reduction was estimated according to Matthis and Erman (1995) using an extinction coefficient (ε) of 27.7 mM^–1^ cm^–1^. To measure CcP activity, the reaction mixture (500 μl) contained 10 mM potassium phosphate pH 7.0, 25 mM ferrocytochrome *c*, and 50 μg protein extract. The reaction was started by adding 200 mM H_2_O_2_. The enzyme assay was performed by measuring the oxidation of ferrocytochrome *c* every 10 s for 3 min.

## Results

### NaCl Activates Osmotic Stress Response

#### Effect on mRNA Level of Genes Coding for Potassium Transporters and Compatible Solutes Biosynthesis

In order to evaluate the adaptive response *of L. ferriphilum* DSM 14647 upon osmotic stress, we determined the mRNA levels of key genes associated with K^+^-uptake, and compatible solutes synthesis and uptake. Total RNA was isolated from cells exposed during indicated times to 100 mM NaCl, a value that represent about 50% of the minimal inhibitory concentration of NaCl for this strain (Rivera-Araya et al., 2017). The RNA levels of the housekeeping gene *rrsB* were quantified as internal expression control.

**FIGURE 1:**
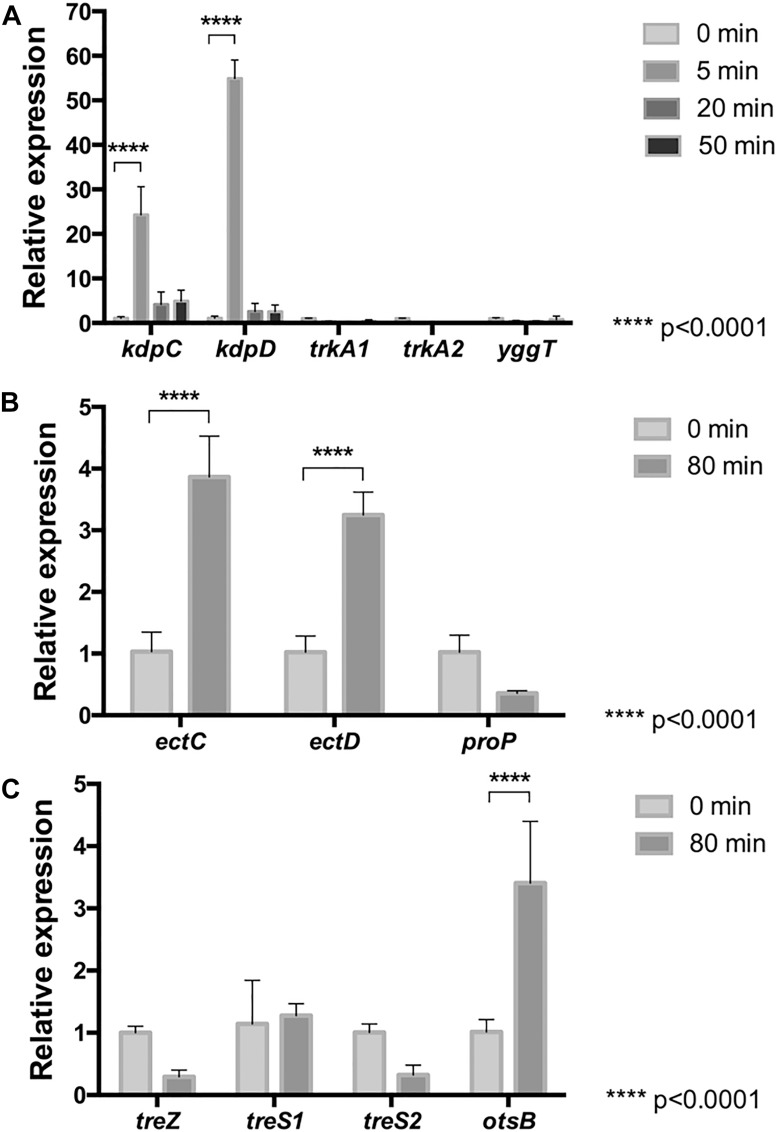
Relative mRNA levels of osmotic stress related genes in *L. ferriphilum* DSM 14647. Relative expression of K^+^ transporter genes was evaluated **(A)** in cells treated with 100 mM NaCl for 5, 20, and 50 min. Relative expression of genes for biosynthesis of (hydroxy)ectoine **(B)** and trehalose **(C)** was evaluated in cells treated with 100 mM NaCl for 0 and 80 min. Data were normalized by the 16S rRNA. Data represent the average of three independent experiments (bars indicate the value range). Statistical analysis was carried out by ANOVA and *t*-Test.

Genes for transporters of potassium (KdpABCD, TrkA, and YggTS) and (hydroxy)ectoine (ProP), and biosynthesis of (hydroxy)ectoine (EctABCD, ProP), and trehalose (TreXYZ, TreS, and OtsAB) have previously been reported as present within the genome of *Leptospirillum* spp. (Rivera-Araya et al., 2017). Thus, to assess relevance of the former, the mRNA level of genes encoding for KdpC, KdpD, TrkA1, TrkA2, and YggT were quantified in cells treated with NaCl for 0, 5, 20, and 50 min. The real-time PCR data showed that *trkA* and *yggT* genes were, in fact, transcribed in this bacterium exposed to NaCl, but they did not show significant changes in their transcript level between treated and untreated cells (Figure 1A). However, the *kdpC* and *kdpD* genes encoding for the Kdp-transport system were significantly (*p* < 0.0001) up-regulated in response to NaCl-induced stress at 5 min of exposure. The increases in the *kdpC* and *kdpD* genes were 25- and 50-fold at 5 min, respectively. On the contrary, at 20 and 50 min of stress the mRNA levels of the *kdpCD* genes were again decreased to normal conditions. These results suggest that in strain DSM 14647 potassium is important to balance the osmotic pressure under NaCl shock as a first response, and the KdpCD transporter seems to play a key role in K^+^ uptake from the medium.

Accumulation of compatible solutes are part of the late-response mechanism to saline stress in microorganisms (Wood, 2015). To evaluate whether this response is also present in *L. ferriphilum*, the mRNA levels of genes associated to uptake (*proP*) and synthesis (*ectC*, *ectD*, *treZ*, *treS1*, *treS2*, and *otsB*) of these compounds from cells exposed to NaCl for 0 and 80 min were measured. As is shown in Figures 1B,C, although the mRNA levels of the encoding gene for the non-specific (hydroxy)ectoine transporter (ProP) tended to decrease, the data analysis showed no significant difference between stressed and non-stressed cells. On the other hand, when mRNA levels of the *ectC* and *ectD* genes encoding for ectoine synthase (EctC) and ectoine hydroxylase (EctD), respectively, were measured (Figure 1B), a significant 4-fold increase was observed, suggesting that ectoine and/or hydroxyectoine are used by *L. ferriphilum* to keep the internal osmotic pressure high under prolonged saline stress.

The mRNA levels of genes coding for key enzymes from each of the three described trehalose synthesis pathways were also evaluated in cells exposed to 100 mM NaCl for 0 and 80 min. The studied genes code for malto-olygosil trehalose trehalohydrolase (*treZ*), trehalose synthase (*treS1*, *treS2*) and trehalose-6-phosphate phosphatase (*otsB*). The results showed that the *treZ* and *treS* genes are expressed in this bacterium under the tested experimental conditions (Figure 1C), tough significant differences between cells from treated and untreated conditions were not detected. Interestingly, as is shown in Figure 1C, the *otsB* gene resulted to be significantly upregulated at 80 min of stress exposure as compared to the control, suggesting that this bacterium synthesizes trehalose by the Ots pathway under the stress conditions studied here. Altogether, these results suggest that *L. ferriphilum* develops a cell-safety mechanism when exposed to long-term osmotic stress, leading to the biosynthesis of (hydroxy)ectoine and trehalose.

#### NaCl Favors Accumulation of Compatible Solutes Hydroxyectoine and Trehalose

**FIGURE 2:**
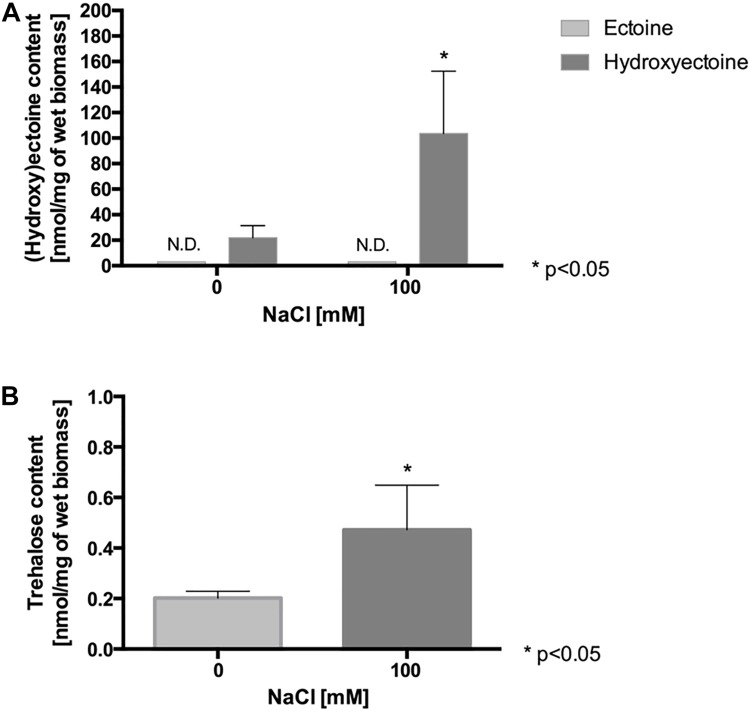
Effect of NaCl exposure on intracellular content of compatible solutes in *L. ferriphilum* DSM 14647. Content of ectoine and hydroxyectoine **(A)**, and trehalose **(B)** in cells exposed to 100 mM NaCl for 90 min. The data represent the average of 3 independent experiments (bars indicate the value range). Statistical analysis was carried out by ANOVA and *t*-Test. N.D.: not detected.

Since (hydroxy)ectoine and trehalose synthesis genes were up-regulated in *L. ferriphilum* exposed to NaCl, the intracellular concentrations of the compatible solutes ectoine, hydroxyectoine and trehalose were monitored. To quantify the compatible solutes, the cells were cultivated in DSMZ 882 medium in the presence of 100 mM NaCl for 90 min. After that, the cells were harvested, and the compatible solutes were extracted and then analyzed by HPLC (ectoine, hydroxyectoine) or GC-MS (trehalose), as described in “Materials and Methods”. As is shown in Figure 2A, ectoine was not detected, however, the hydroxyectoine was detected in both treated and untreated cells. Under control conditions, the intracellular content of hydroxyectoine was 20 nmol/mg wet biomass. Interestingly, when the cells were exposed to NaCl, the intracellular content of this compatible solute significantly increased to 100 nmol/mg wet biomass. On the other hand, the intracellular trehalose content also increased under salinity conditions (100 mM NaCl), reaching a concentration of 0.47 nmol/mg wet biomass, 2.3-fold the content of this molecule under control conditions (Figure 2B). These results showed that the concentrations of hydroxyectoine and trehalose are clearly salt dependent, and that hydroxyectoine is the major compatible solute for *L. ferriphilum*, while trehalose would be a minor component.

### NaCl Exposure Decreases the Intracellular pH (pH_in_)

**FIGURE 3:**
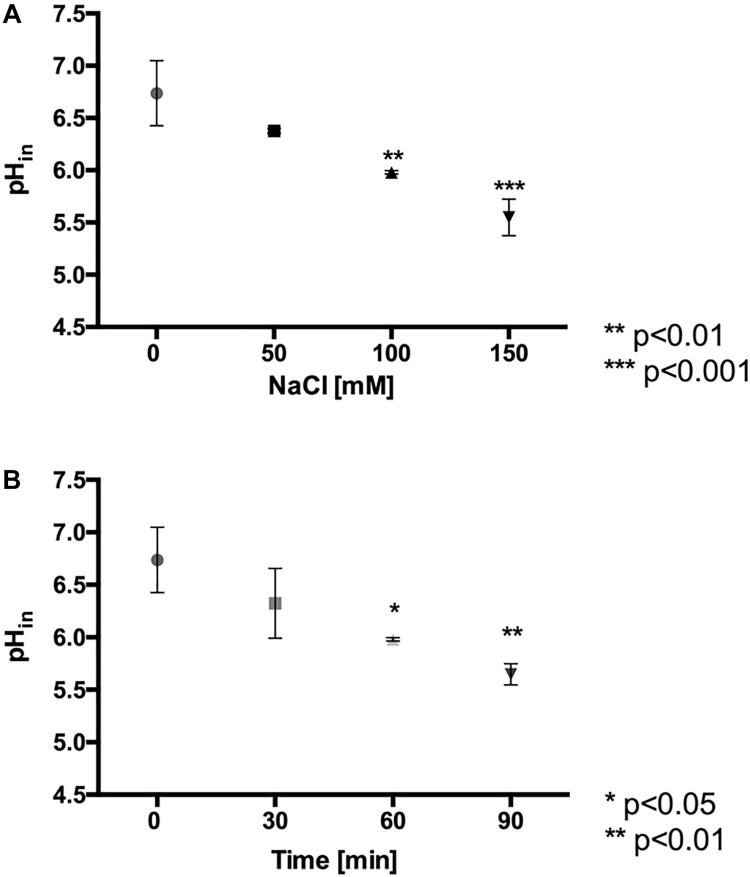
Effect of NaCl exposure on intracellular pH (pH_in_) in *L. ferriphilum* DSM 14647. Effect of NaCl concentration on pH_in_ of cells exposed for 60 min to the stress condition **(A)**. Effect of the exposure time in cells exposed to 100 mM NaCl **(B)**. The data represent the average of 3 independent experiments (bars indicate the value range). Statistical analysis was carried out by ANOVA Test.

It has been described that the presence of anions in the culture medium of the acidophiles *At. ferrooxidans* and *At. thiooxidans* reduces their iron oxidation activity and their intracellular pH (pH_in_) (Alexander et al., 1987; Suzuki et al., 1999). Thus, it raised the question whether there is a similar effect when *L. ferriphilum* is exposed to high chloride conditions. According to Figure 3, these cells keep an intracellular pH of 6.7 under standard culture conditions (pH_medium_ = 1.8). However, when the cells were exposed to 100 and 150 mM NaCl, the intracellular pH significantly decreased to close to 6.0 (*p* < 0.01) and 5.5 (*p* < 0.001), respectively (Figure 3A). In addition, as shown in Figure 3B, the exposure of the cells to 100 mM NaCl for different times showed that at 60 and 90 min, the pH_in_ was significantly decreased approximately 6.0 (*p* < 0.05) and 5.7 (*p* < 0.01), respectively. As expected, control cells that was kept during 90 min in fresh medium without NaCl stress did not show changes in the estimated pH values, so an effect of the probe should be ruled out. These results suggest that the chloride ion induces a similar effect in *L. ferriphilum* as in other acidophiles, acidifying the intracellular pH, thus likely impairing cellular function. Finally, it should be mentioned that measurement of intracellular pH required the incubation of the cells for 30 min at neutral pH. Thus, in order to determine whether this incubation affected the viability, cells were exposed to phosphate-buffered saline pH 7.4, and subsequently inoculated into fresh medium and cultured. We observed an extension of the lag phase, thus indicating a pronounced negative effect on physiology, but at the same time showing the preservation of cell viability (Supplementary Figure S1). This aspect will additionally be discussed below.

### NaCl Exposure Induces Oxidative Stress

While acidophilic bacteria can grow in acidic environments, they are able to keep the intracellular pH close to neutrality. The pH-homeostasis in these microorganisms is mainly kept by respiration, because the electron transport chain exports and consumes intracellular protons during its activity. However, one of the most severe effects of chloride exposition is the cytoplasm acidification, a fact that, as has been shown here, also occurs in *Leptospirillum* sp. Thus, in order to bring back the intracellular pH close to neutrality, acidophiles could increase the activity of the respiratory chain and consequently, oxygen consumption. Additionally, the increase in the respiratory rate might favor the production of reactive oxygen species (ROS). In order to evaluate whether chloride ions favor ROS production by an increase in the respiratory rate in *L. ferriphilum* DSM 14647, we measured the oxygen consumption, the intracellular total ROS production and the activity of antioxidant proteins upon saline stress.

#### NaCl Exposure Increases the Respiratory Rate

**FIGURE 4:**
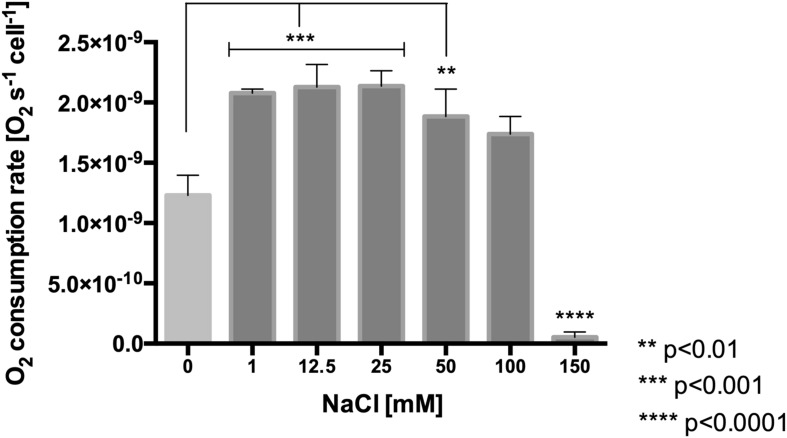
Effect of NaCl concentration on oxygen consumption rate in *L. ferriphilum* DSM 14647. The data represent the average of three independent experiments (bars indicate the value range). Statistical analysis was carried out by ANOVA Test.

To evaluate the effect of NaCl on the respiratory rate, the oxygen consumption rate was estimated in cells exposed to a range of 0–150 mM NaCl for 30 min. As is shown in Figure 4, the values of oxygen consumption proved to be significantly higher (from 1.2 × 10**^–^**^9^ to 2 × 10**^–^**^9^ s**^–^**^1^cell**^–^**^1^, *p* < 0.001) in cells exposed to range of 1–50 mM NaCl, as compared to the control condition. Interestingly, at 150 mM NaCl only an extremely low respiration rate was observed, indicating that this might be a concentration which *L. ferriphilum* cannot cope with.

#### NaCl Induces ROS Production

**FIGURE 5:**
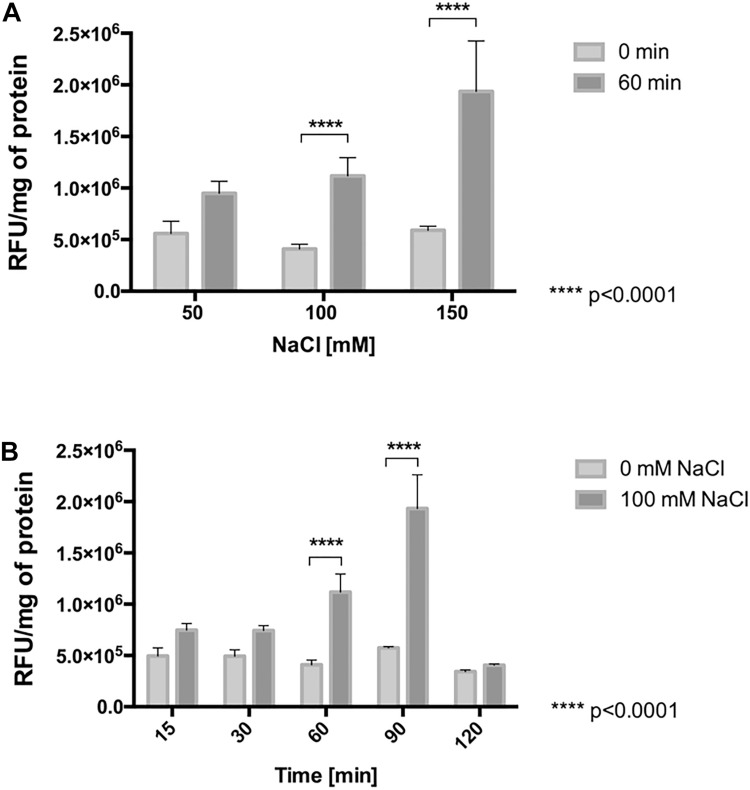
ROS generation in *L. ferriphilum* DSM 14647. Effect of NaCl concentration **(A)** and exposure time **(B)** on intracellular ROS generation in the bacteria. The concentration effect was evaluated in cells exposed to NaCl for 60 min. The effect of exposure time was evaluated in cells exposed to 100 mM NaCl. Cytoplasmic ROS content was expressed as relative fluorescence units (RFU) of the activated fluorescent probe H_2_DCFDA per mg of protein.

To determine whether the increase in the respiratory rate, resulting from chloride stress induces an increase of reactive oxygen species (ROS), the intracellular content of ROS was measured in cells treated with NaCl and compared to the content in non-stressed control cells. As shown in Figure 5A, with mere presence of 50 mM NaCl for 60 min in *L. ferriphilum*, the intracellular content of ROS started to increase compared with the control. Interestingly, cells exposed to 100 and 150 mM NaCl for 60 min showed significantly increased ROS content (to 274 and 328%, respectively) as compared to control cells (100%). Additionally, Figure 5B shows that cells exposed to 100 mM NaCl for 90 min also had a significantly increased ROS content of 336% compared with control cells. Altogether, these results suggest that the presence of NaCl in the culture medium produces a severe oxidative condition in cells of *L. ferriphilum* DSM 14647.

Since cobalamin (vitamin B12) has a suppressive antioxidant effect on total ROS generation in *Leptospirillum* strain CF-1 (Ferrer et al., 2016b) and since the compatible solute hydroxyectoine protects biomolecules from high osmolarity conditions and maintains their native functions (Widderich et al., 2014), to get additional insights into the response to NaCl, we studied the effect of the external supplementation of cobalamin and hydroxyectoine in the culture medium of *L. ferriphilum* on ROS generation. To evaluate the effect of cobalamin, the culture was pre-treated with 5 nM of this vitamin for 60 min and, after that, treated with 100 mM NaCl for 90 min. As expected, NaCl exposure led to a significant increase in ROS generation (254%), compared with cells grown under control conditions (100%). Interestingly, pre-treatment with cobalamin significantly reduced the ROS level from 254% (in NaCl-stressed cells) to 137% (in NaCl-stressed and cobalamine pre-treated cells), a level that is comparable to those observed under control conditions without stress (100%) (Supplementary Figure S2A).

To evaluate the effect of hydroxyectoine, the culture exposed to 100 mM NaCl for 90 min was treated with 0.5 mM of this compound. The results showed that NaCl-stressed cells had a significantly increased ROS level (240%). However, the stressed cells treated with hydroxyectoine had a decreased ROS production (155%, *p* < 0.05) compared to the stressed cells not treated with the compatible solute (Supplementary Figure S2B). These results suggest that a relation between chloride stress and the generation of oxidative stress by NaCl may exist in acidophilic microorganisms, since the stress may partially be alleviated with an antioxidant or a compatible solute.

#### NaCl Increases the Activity of Protective Antioxidant Proteins

**FIGURE 6:**
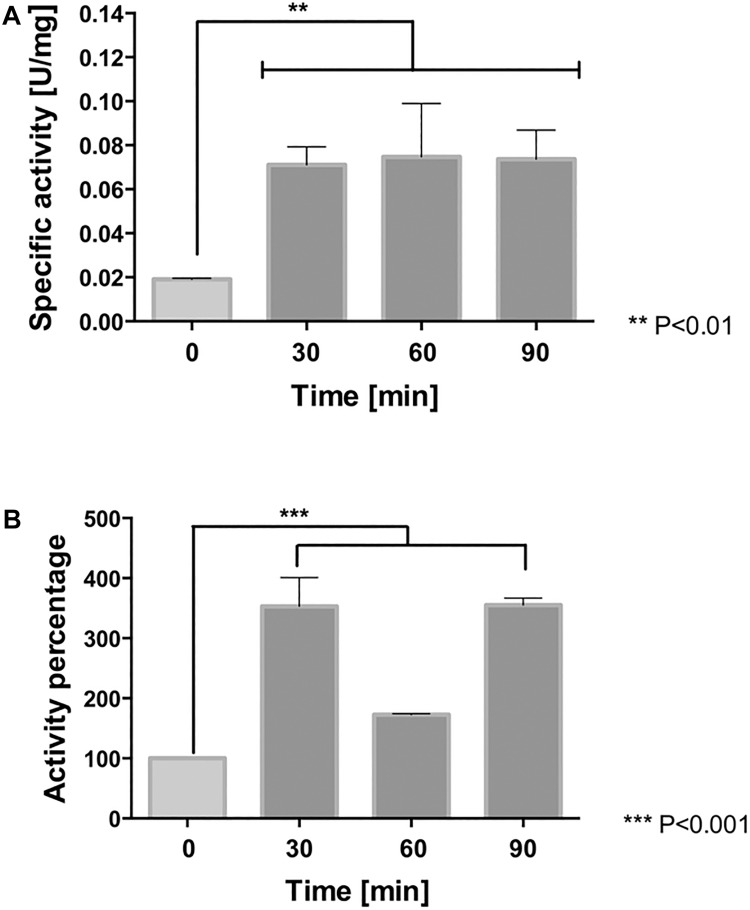
Antioxidant protein activity in *L. ferriphilum* DSM 14647. The cytochrome *c* peroxidase **(A)** and thioredoxin activity **(B)** in cells exposed to 100 mM NaCl. The data represent the average of three independent experiments (bar indicates the value range). Statistical analysis was carried out by ANOVA Test.

As previously described, *Leptospirillum* spp. activates peroxidase and thioredoxin-based thiol/disulfide systems to face oxidative stress (Norambuena et al., 2012; Contreras et al., 2015; Zapata et al., 2017). In order to determine whether NaCl-induced ROS was accompanied by an increase in activity of antioxidant proteins, the activities of cytochrome *c* peroxidase (CcP) and of thioredoxins (Trx) were measured in whole cell extracts derived from cells exposed to 100 mM NaCl for 0, 30, 60, and 90 min. As is shown in Figure 6A, at 30, 60, and 90 min of exposure to NaCl, the cytochrome *c* peroxidase activity resulted significantly higher (4-fold) compared to the control (*p* < 0.01). The exposure of the cells to chloride stress also led to an increase (3.5-fold, *p* < 0.001) in thioredoxin activity at 30 and 90 min. However, this activity appeared declined at 60 min (1.5-fold) compared to the control conditions (Figure 6B). It is likely part of the natural redox cycle of thioredoxins that occurs during the antioxidant response and which, in turn, is dependent on the activity of thioredoxin reductase (Norambuena et al., 2012). These results clearly indicate that peroxidase and thioredoxin systems are activated as part of the antioxidant response of *L. ferriphilum* DSM 14647 to fight against severe oxidative conditions induced by chloride exposure.

Additionally, we also evaluated the effect of cobalamin and hydroxyectoine on the activity of antioxidant proteins. Interestingly, the pre-treatment with 5 nM cobalamin led to a significant additional increase of both CcP (613%) and Trx (527%) activity in cells exposed to NaCl compared to activity measured in salt-exposed cells, but not cobalamin-pretreated (CcP: 317% and Trx: 362%) (Supplementary Figures S2C,D). On the contrary, the treatment with 0.5 mM hydroxyectoine led to a decreased activity of CcP (160%) and Trx (218%) in cells exposed to NaCl stress compared to stressed cells that were not treated with this compatible solute (CcP: 317% and Trx: 362%) (Supplementary Figures S2C,D). Thus, although cobalamin and hydroxyectoine can significantly reduce the intracellular ROS level under chloride stress, according to these results they would exert their antioxidant effect by mechanisms operating at different levels of the ROS control machinery.

#### NaCl Increases the mRNA Level of Genes Encoding for Antioxidant Proteins

**FIGURE 7:**
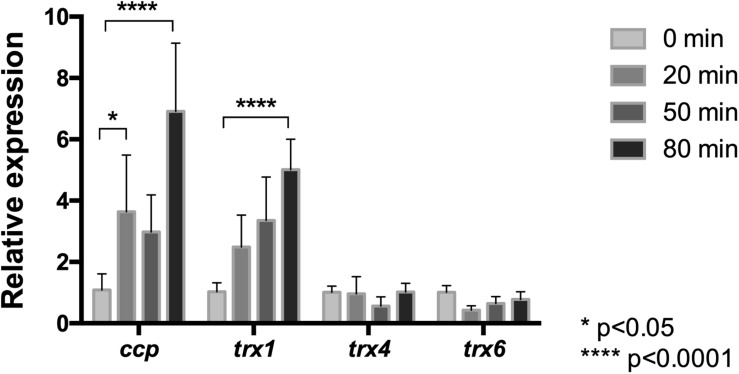
Relative mRNA levels of oxidative stress related genes in *L. ferriphilum* DSM 14647. Relative expression of cytochrome *c* peroxidase (*ccp*) and the thioredoxin (*trx*) encoding genes was evaluated in cells treated with 100 mM NaCl. Data were normalized by the *rrsB* gene. Data represent the average of three independent experiments (bars indicate the value range). Statistical analysis was carried out by ANOVA Test.

Since chloride-mediated stress results in increased activity of cytochrome *c* peroxidase and thioredoxins of *L. ferriphilum* DSM 14647, the transcriptional response of this bacterium under NaCl exposure was assessed. We analyzed the mRNA level profiles of *ccp*, *trx1*, *trx4*, and *trx6* encoding for CcP (Zapata et al., 2017), and Trxs (Norambuena et al., 2012), respectively. The expression level of each gene was quantified at 0, 20, 50, or 80 min after exposure to the chloride stress condition using RT-qPCR assays. As is shown in Figure 7, the mRNA level of the *ccp* gene was up-regulated at all the assayed times, but it resulted to be significantly higher than the control at 20 and 80 min (*p* < 0.05 and *p* < 0.0001, respectively). The expression profile of the *ccp* gene is in agreement with the detected increased activity of the CcP enzyme described above.

Concerning to the thioredoxin encoding genes, the obtained results (Figure 7) showed that, although mRNA levels from the *trx1*, *trx4*, and *trx6* genes were detected in cells exposed to 100 mM NaCl at all exposure times, only the mRNA level of *trx1* increased significantly (5-fold) in response to chloride stress compared to the control conditions (*p* < 0.0001). It is interesting to note that the detected increase of the *trx1* gene was in agreement with the detected increase of Trx activity in cells exposed to NaCl (Figure 6B). Altogether, the expression profiles of the *ccp* and *trx1* genes suggest that oxidative stress induced by NaCl triggers a transcriptional response that contributes to increase the activity of antioxidant proteins as a strategy to endure the oxidative conditions.

## Discussion

Acidophilic sulfur- and iron-oxidizing bacteria have been described as very sensitive to the presence of anions, in particular chloride. Until now, the reasons of this have been ascribed mainly to two aspects: the osmotic imbalance and the acidification of the cytoplasm (Zammit and Watkin, 2016). Here, we propose that as a consequence of a decrease in the intracellular pH and in order to restore the pH homeostasis, the cells respond by increasing the respiratory rate which could favor generation of ROS. Thus, in this article, a multifactorial effect of chloride is proposed, and parameters to evaluate osmotic imbalance, cytoplasmic acidification, and oxidative stress in *L. ferriphilum* DSM 14647 upon NaCl exposure were analyzed.

When studying the osmoprotection mechanism, our results showed that the exposition of *L. ferriphilum* to NaCl produced osmotic stress, as judged by transcription of genes related with canonical osmoregulation. Such that, according to the characteristics of a first response against saline stress described for neutrophilic and halophilic microorganisms (Csonka, 1989; Kempf and Bremer, 1998; Krämer, 2010; Wood, 2015; Kindzierski et al., 2017), the mRNA level of two genes associated with the regulated K^+^ uptake system Kdp exhibited a significant increase immediately after exposure to NaCl. Additionally, in a longer-term response, the expression of *kdp* genes was again reduced and instead the transcription level of genes associated with *de novo* biosynthesis of (hydroxy)ectoine and trehalose showed a significant increase, suggesting the up-regulation of the EctABCD and OtsAB pathways. In agreement with these facts, after exposure to NaCl hydroxyectoine and trehalose exhibited significantly enhanced intracellular content. Interestingly, hydroxyectoine is preferentially produced over ectoine and trehalose in *L. ferriphilum* DSM 14647. In agreement with these results, an early meta-transcriptomic analysis performed in acidic water from Rio Tinto (Spain), dominated by *L. ferrooxidans*, showed that the gene clusters *ectABCD*, *otsAB* and *kdpABCD* were up-regulated in a place with high presence of NaCl (Parro et al., 2007). Additionally, previous proteomic studies showed that in AMD biofilms with *Leptospirillum* group II bacteria, there was a high production of proteins necessary for ectoine and hydroxyectoine biosynthesis (Goltsman et al., 2009; Belnap et al., 2010; Mosier et al., 2013). Moreover, Mosier et al. (2013) detected the presence of only hydroxyectoine and not ectoine in the AMD biofilm by stable isotope labeling coupled with HPLC. For other organisms, early studies have suggested that the hydroxylation of ectoine has always been linked to extreme conditions, as this may be a survival strategy (García-Estepa et al., 2006; Van-Thuoc et al., 2013; Tao et al., 2016), which is consistent with the findings in our study. Despite their closely related chemical structures, 5-hydroxyectoine often possesses a superior protective effect than its precursor ectoine (Van-Thuoc et al., 2013). Therefore, hydroxyectoine may be the main osmolyte against osmotic stress under the extreme conditions encountered in acidic saline environments.

Measurements of intracellular pH (pH_in_) of *L. ferriphilum* DSM 14647 performed in this work, showed a significant decrease of pH_in_ upon exposure to NaCl and the effect was even a function of the time of exposure and of salt concentration. We acknowledge that the incubation of the cells at pH 7.4 for 30 min, which was necessary to allow the fluorescence dye pHrodo^TM^ Green to enter the cells, may have represented an additional stress for the cells. However, the control cells (not exposed to NaCl) were treated in the same way, so that it is clear that the effect seen is due to NaCl and not to the incubation at pH 7.4. In addition, there is no way that the incubation at pH 7.4 could have selectively acidified the intracellular pH to 6.0 or even 5.5. The results agree with previous report for *At. ferrooxidans*, where exposure of this bacterium to 100 mM NaCl for 1 h reduced the cytoplasmic pH to close to 5.1 (Alexander et al., 1987). The acidification of the cytoplasm in presence of NaCl is predicted to be a consequence of a massive chloride entry which would be favored by the positive membrane potential. A drop in the membrane potential due to the movement of anions will favor the entry of protons and concomitant acidification (Alexander et al., 1987; Zammit and Watkin, 2016). In acidophiles, the effect of chloride is exacerbated by the high external proton concentration in the medium and the large pH gradient over the cellular membrane. Thus, it is possible to predict that the toxic effect of chloride is inversely dependent on the external pH. This relationship was, in fact, described for *Acidithiobacillus thiooxidans*, where permeability to chloride and its toxicity were shown to increase as the external pH decreases (Suzuki et al., 1999). A similar effect was also detected in *L. ferriphilum* DSM 14647, since the change of pH of the media from 4 to 1.8 had a dramatic effect on minimal inhibitory concentration (2.3 folds lower), and therefore, on the tolerance of this bacterium (data not published). Finally, the effect of lowering the intracellular pH suggests that during chloride stress the cell must also face a situation of acidic stress and therefore, the mechanisms that facilitate pH homeostasis in acidophiles could play an important role in salt tolerance. Thus, although some studies have approached this topic (Mangold et al., 2013), more efforts should be made to understand the mechanisms that allow tight control and maintenance of intracellular pH (around 6.5) in microorganisms that inhabit extremely acidic environments (pH < 3).

As predicted, exposure of *L. ferriphilum* to chloride stress significantly increases the respiratory rate, as deduced from oxygen consumption values. In addition, cells showed a significant increase in the intracellular ROS level, suggesting that a generalized oxidative condition has been elicited by chloride exposure. The detected increase in ROS content is due to the increase in the rate of oxygen consumption and the predicted increase in the activity of the electron transport chain between Fe (II) and O_2_. However, the cell is also under a strong condition of osmotic and pH stresses that could damage macromolecules, favoring the release of metal ions from metalloproteins abundant in these microorganisms (Yarzábal et al., 2002; Potrykus et al., 2011; Levicán et al., 2012). The relationship between osmotic and pH stresses in the induction of redox stress has previously been reported for neutrophilic microorganisms (Wilks et al., 2009; Mols et al., 2010).

In agreement with the accumulation of intracellular ROS, real time RT-PCR and protein activity assays showed that the antioxidant protein activities CcP and Trx were elevated upon saline exposure. The increase in mRNA levels of the corresponding *ccp* and *trx* genes suggests that, at least in part, this activation is supported at the transcriptional level. In general terms, these results are consistent with increased oxygen consumption reported for *Acidithiobacillus thiooxidans* upon exposure to the chloride salts KCl, NaCl, and LiCl exposure (Suzuki et al., 1999). In the same line, a transcriptomic analysis performed in *Acidimicrobium ferrooxidans* under saline stress with 100 mM NaCl revealed the up-regulation of a peroxidase encoding gene (Zammit et al., 2012). Furthermore, a proteomic analysis carried out in *Acidithiobacillus caldus* exposed to 500 mM NaCl showed up-regulation of a gene for a thiol-peroxidase (Guo et al., 2014). Additionally, a proteomic study showed that *Ac. prosperus* over-expressed rubrerythrin and Dyp-type peroxidases upon saline stress induction (Dopson et al., 2017). Thus, these findings suggest that in acidophilic microorganisms, saline stress triggers a cellular antioxidant response that mainly involves the activation of proteins for the scavenging of inorganic peroxide, thereby avoiding Fenton chemistry and the generation of highly deleterious hydroxyl radical (Ferrer et al., 2016a). According to our results, *L. ferriphilum* DSM 14647 displays a similar response that involves activation of cytochrome *c* peroxidase activity. However, the system in charge of the thiol/disulfide balance also seems to have a role in the maintenance of redox homeostasis in this bacterium under NaCl stress.

An interesting finding of this work was that the external supplementation of an antioxidant or compatible solute such as cobalamin or hydroxyectoine, respectively, attenuates chloride-induced ROS accumulation. These compounds do, however, have a totally opposite effect on the activity of antioxidant proteins of strain DSM 14647. The external addition of cobalamin to the cell culture clearly increased the activity of the antioxidant proteins CcP and Trx, while the addition of hydroxyectoine did not exert any significant effect on the activity of these proteins. As previously described (Ferrer et al., 2016b), the effect of cobalamin involves the activation of antioxidant proteins that will subsequently exert an effect by reducing the level of reactive oxygen species. For its part, hydroxyectoine exerts a protective effect on biomolecules in response to osmotic stress. It can be predicted that during chloride-induced stress, the uptake and accumulation of this compatible solute attenuated the release of metals from metalloproteins and so also the generation of ROS in the cytoplasm. In addition, at potential effect of hydroxyectoine to reduce ROS through other mechanisms, like a direct antioxidant activity, cannot be excluded (Sajjad et al., 2018). Altogether, these data give us a wider view about how operating mechanisms supporting different adaptive responses can contribute to regulate cytoplasmic ROS concentration. At the same time, these results pointed out the fact that chloride exposure can induce different stresses to the cell, and the way in which the cell responds to all of them as a whole will be a determining factor of NaCl tolerance in acidophilic microorganisms.

It is important to remark that although we have provided strong evidences that support a view where osmotic, acidic and oxidative stresses are induced in presence of NaCl, additional factors explaining the high toxicity of chloride could be still unveiled. In *Leptospirillum* sp., like other acidophilic microorganisms, basic proteins with high pI values are abundant in the surface as a way to avoid protons entrance, while the intracellular proteins remain close to neutrality (Mangold et al., 2011). In contrast, in the moderate halophilic bacterium *Halobacillus halophilus* a high percentage of acidic proteins with low pI values have been described as an unusual strategy that allows this bacterium to accumulate and to tolerate high concentration of chloride (Saum et al., 2012). Thus, based on the pI′s profile, we predict that the proteins from acidophiles could be particularly more sensitive to the denaturing effect of chloride. Whether a general relation between pI of proteins and anion tolerance in microorganisms exist should be approached.

**FIGURE 8:**
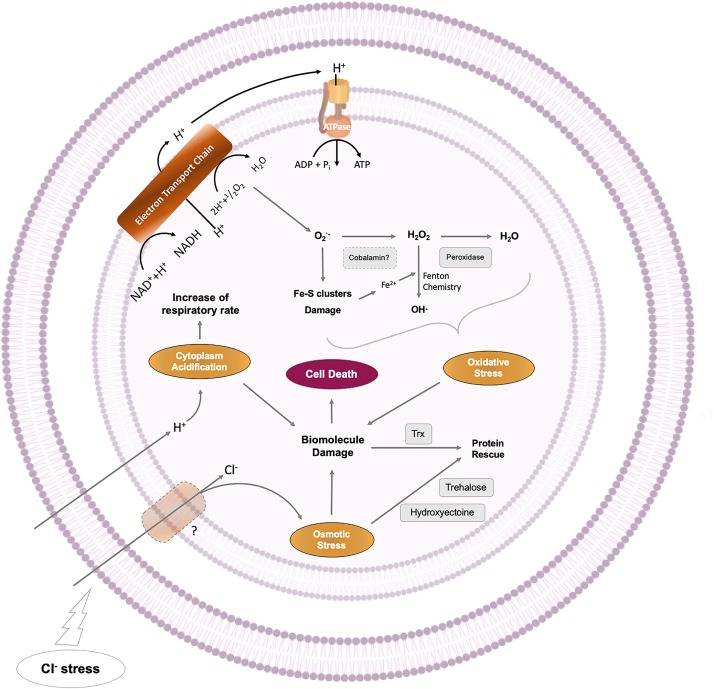
Multifaceted effect of NaCl in *L. ferriphilum* DSM 14647.

Finally, based on the obtained results is possible to better understand the multifactorial effect of chloride-stress in acidophilic bacteria, and therefore decipher the molecular basis of the extreme sensitivity of these microorganisms to chloride and other anions. We propose a model for chloride-induced effects on *Leptospirillum* DSM 14647 (Figure 8). This understanding provides a potential approach to enhance the adaptation of acidophilic iron-oxidizing microorganisms to industrial bioleaching processes.

## Data Availability Statement

All datasets generated for this study are included in the article/Supplementary Material.

## Author Contributions

JR-A and GL designed the research. JR-A, DH, AP, and RC performed the experimental and bioinformatics research, and analyzed the data. JR-A, MS, and GL prepared the manuscript.

## Conflict of Interest

The authors declare that the research was conducted in the absence of any commercial or financial relationships that could be construed as a potential conflict of interest.
