# Supplementary figures and images for: Osmotic Imbalance, Cytoplasm Acidification and Oxidative Stress Induction Support the High Toxicity of Chloride in Acidophilic Bacteria
Source: Front Microbiol. 2019 Oct 29;10:2455. doi: 10.3389/fmicb.2019.02455 (PMC6828654; doi:10.3389/fmicb.2019.02455)

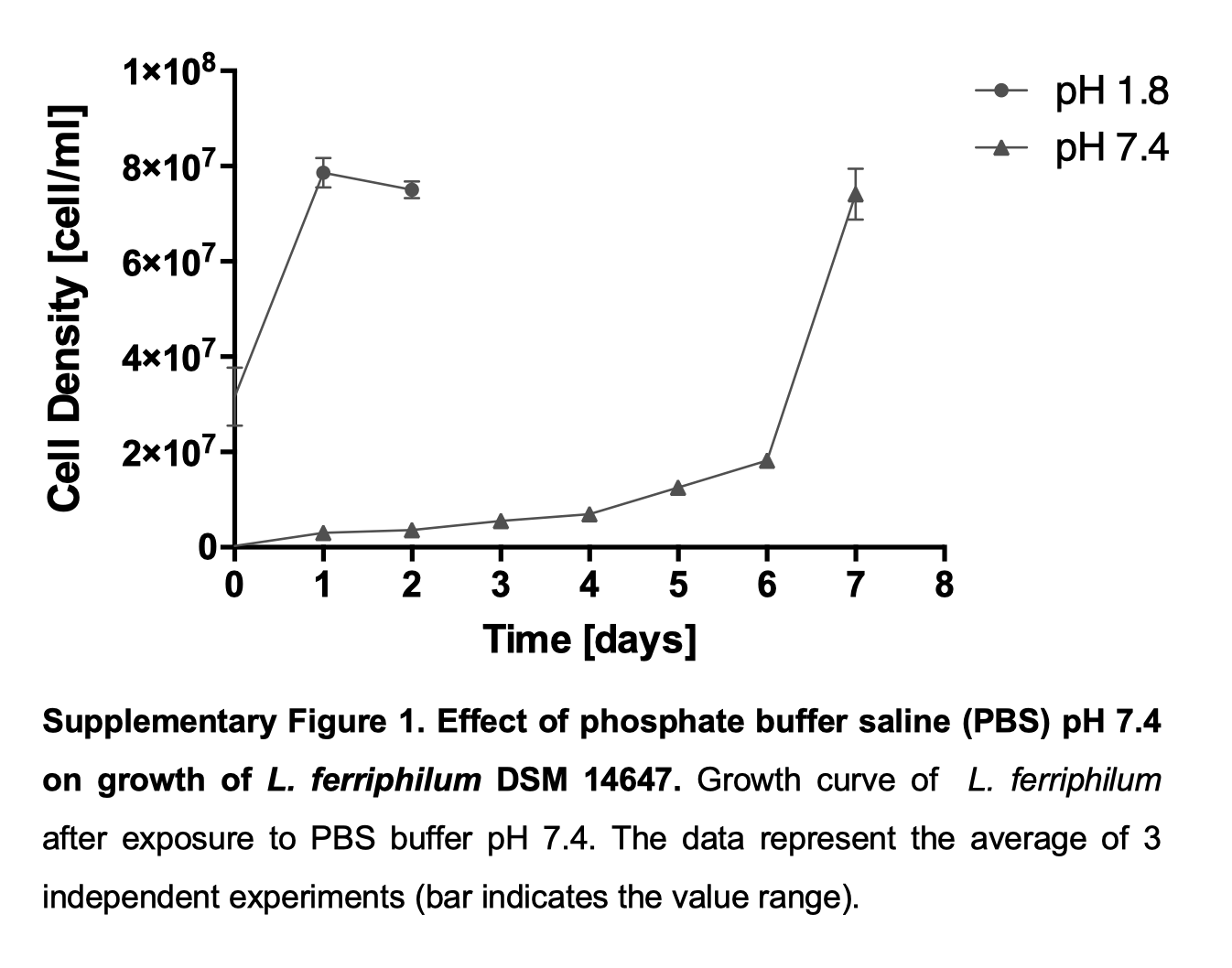

Supplement: Supplementary file 1 [file Image_1.PNG]

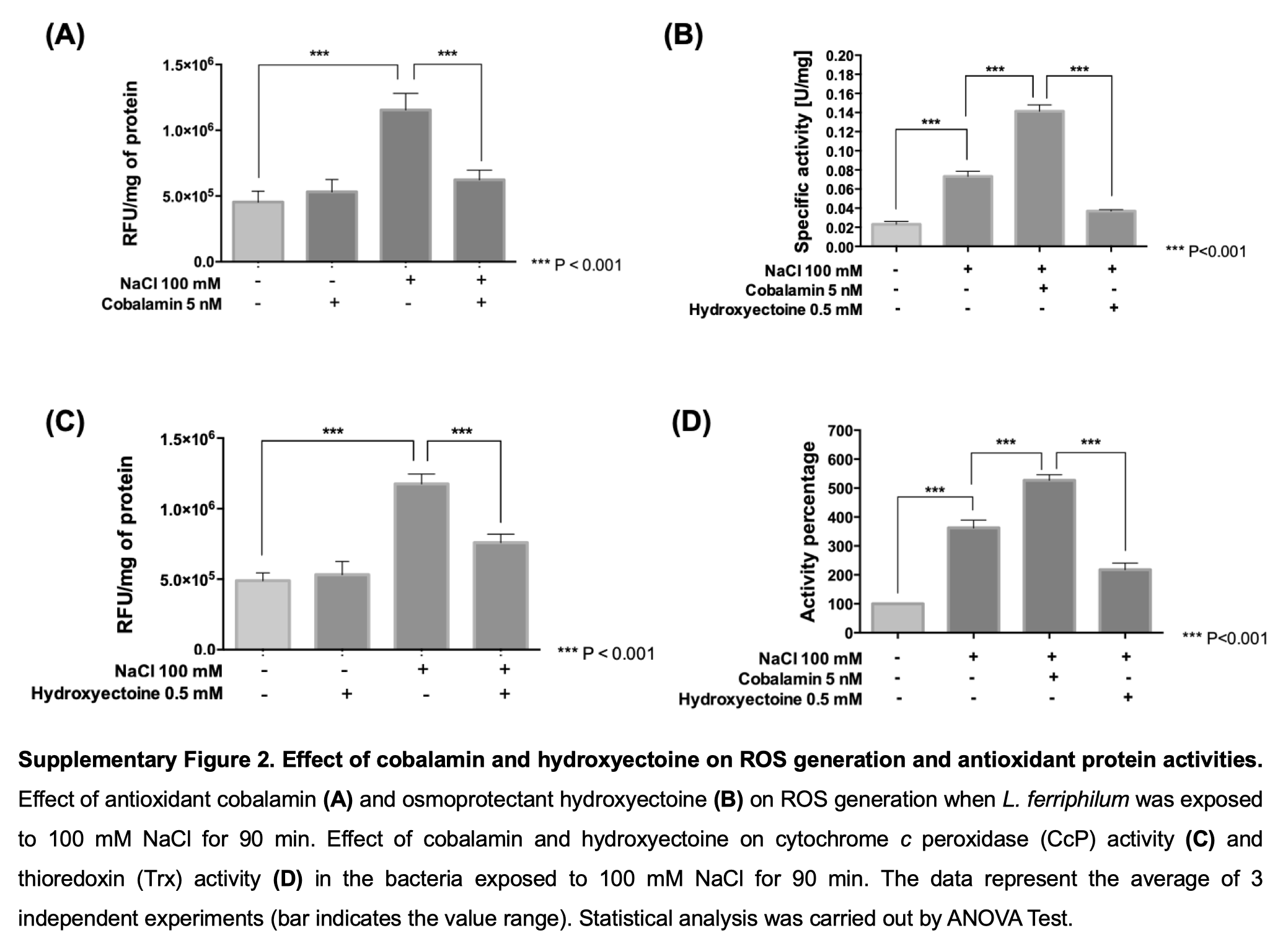

Supplement: Supplementary file 2 [file Image_2.png]
